# The application of metagenomic next-generation sequencing in diagnosing *Chlamydia psittaci* pneumonia: a report of five cases

**DOI:** 10.1186/s12890-020-1098-x

**Published:** 2020-03-17

**Authors:** Lei Gu, Wei Liu, Meng Ru, Jing Lin, Guoqing Yu, Jia Ye, Zheng-an Zhu, Yuebin Liu, Jian Chen, Guoxiang Lai, Wen Wen

**Affiliations:** 1 Graduate College of Fujian Medical University, Minhou, Fuzhou, 350108 China; 2 Department of Respiratory and Critical Care Medicine, Dongfang Hospital of Xiamen University, Fuzhou General Hospital of Fujian Medical University, The 900th Hospital of the Joint Logistic Support Force, PLA, Gulou, Fuzhou, 350025 China; 3 Pharmacy Department, The 985th Hospital of the Joint Logistic Support Force, PLA, Yingze, Taiyuan, 030001 China; 4 Department of Nephrology, Dongfang Hospital of Xiamen University, Fuzhou General Hospital of Fujian Medical University, The 900th Hospital of the Joint Logistic Support Force, PLA, Gulou, Fuzhou, 350025 China

**Keywords:** mNGS, *Chlamydia psittaci*, Case report

## Abstract

**Background:**

*Chlamydia psittaci* pneumonia is a zoonotic infectious disease caused by *Chlamydia psittaci*. Diagnostic tools, including culture, serologic test and PCR-based methods, are available but prone to false negative results.

**Case presentation:**

This report included five cases of *Chlamydia psittaci* pneumonia. Symptoms and signs common to all 5 cases included fever, coughing, generalized muscle ache, and most notably, inflammatory infiltration of the lungs upon chest CT and X-ray. Metagenomic next-generation sequencing (mNGS) revealed the presence of *Chlamydia psittaci* in biopsy lung tissue in 3 cases and bronchoalveolar lavage fluid in the remaining 2 cases. Three patients responded to doxycycline plus moxifloxacin; two patients responded to moxifloxacin alone.

**Conclusions:**

mNGS could be used to diagnose *Chlamydia psittaci* pneumonia.

## Background

*Chlamydia psittaci* is an obligatory intra-cellular Gram-negative bacterium that typically infects birds, but could occasionally cause psittacosis in humans when contaminated aerosols from infected birds are inhaled. *Chlamydia psittaci* pneumonia in humans is underestimated due to low awareness of the disease and atypical clinical presentation in majority of the cases [1–3]. The low sensitivity and complex procedure of *Chlamydia psittaci* culture causes it hardly routinely performed in most diagnostic laboratories. Other laboratory testing included serological assay and polymerase chain reaction (PCR) based methods, but both have questionable sensitivity and specificity [2]. Untargeted metagenomic next-generation sequencing (mNGS) has been increasingly used in the diagnosis of infectious diseases, particularly when conventional diagnostic approaches have limitations [4]. Here we report 5 cases of *Chlamydia psittaci* pneumonia, in which the diagnosis was established with mNGS. All together, mNGS was conducted in a total of 120 pneumonia cases in the index period. Demographical and basic clinical features of the 5 cases are summarized in Table 1.

**Table 1:** Demographical and basic clinical features of the 5 patients

Case #	Sex	Age (y)	Sample	Underlying diseases	Laboratory test				metagenomics sequencing results and specific reads(n)
					routine blood test	CRP (mg/L)	PCT (ng/ml)	ESR (mm/h)	
**1**	**Female**	**81**	**BALF**	**hypertension, diabetes, coronary artery diseases**	WBC 7.71*10^9, NE% 69.8, NE 5.38*10^9, LN% 17.5↓, LN 1.35*10^9	**53**	**<0.05**	**74**	***Candida albicans*** **(182) Staphylococcus capitis (12)** **Chlamydia psittaci (6) Human betaherpesvirus 5 (2)**
**2**	**Male**	**45**	**BALF**	**diabetes**	WBC 8.22*10^9, NE% 93.1↑, NE 7.65*10^9↑, LN% 4.0↓, LN 0.33*10^9↓	**226**	**1.712**	**42**	**Chlamydia psittaci (225) Lautropia mirabilis (28) Leuconostoc lactis (19)** **Rothia mucilaginosa (19) Rothia dentocariosa (12)** **Streptococcus parasanguinis (10) Actinomyces odontolyticus (8)** ***Streptococcus mitis*** **(4)**
**3**	**Female**	**85**	**Lung tissue**	**none**	WBC 6.38*10^9, NE% 88.9, NE 5.67*10^9, LN% 8.0↓, LN 0.51*10^9	**184**	**1.64**	**64**	**Chlamydia psittaci (48)** ***Staphylococcus epidermidis*** **(1)**
**4**	**Female**	**66**	**Lung tissue**	**none**	WBC 5.47*10^9, NE% 69.8, NE 3.82*10^9, LN% 19.4↓, LN 1.06*10^9↓	**124**	**0.13**	**17**	**Chlamydia psittaci (205) Corynebacterium striatum (1)** ***Klebsiella pneumoniae*** **(1)**
**5**	**Female**	**61**	**Lung tissue**	**none**	WBC 6.88*10^9, NE% 68.4, NE 4.70*10^9, LN% 22.7↓, LN 1.56*10^9↓	**96.7**	**<0.05**	**37**	**Chlamydia psittaci (2)**

*Abbreviations*: *BALF* Bronchoalveolar lavage fluid, *CRP* C-reactive protein (normal reference range: 0–8 mg/L), *ESR* Erythrocyte sedimentation rate (normal < 15 mm/h);

*LN* Lymphocyte, *PCT* Procalcitonin (normal < 0.05 ng/ml), *NE* Neutrophil, *WBC* White blood cell

## Case presentation

### Case #1

An 81-year-old woman was transferred to us with fever, productive coughing with white sticky sputum, and generalized muscle ache and malaise for 10 days. On the 5th day after the onset of symptoms, she visited a local hospital. A chest computed tomography (CT) scan showed inflammatory infiltration in the lower lobe of the left lung. She was treated with moxifloxacin (0.4 g, I.V., qd) plus meropenem (1 g, I.V., q12h) for 3 days, but fever continued (highest body temperature 39.4 °C). A repeat CT scan suggested progression of pulmonary infection. Moxifloxacin was replaced with linezolid (600 mg, I.V., q12h), with continuing meropenem treatment. On the second day after linezolid/meropenem, fever subsided. However, other symptoms and signs continued.

**Fig. 1:**
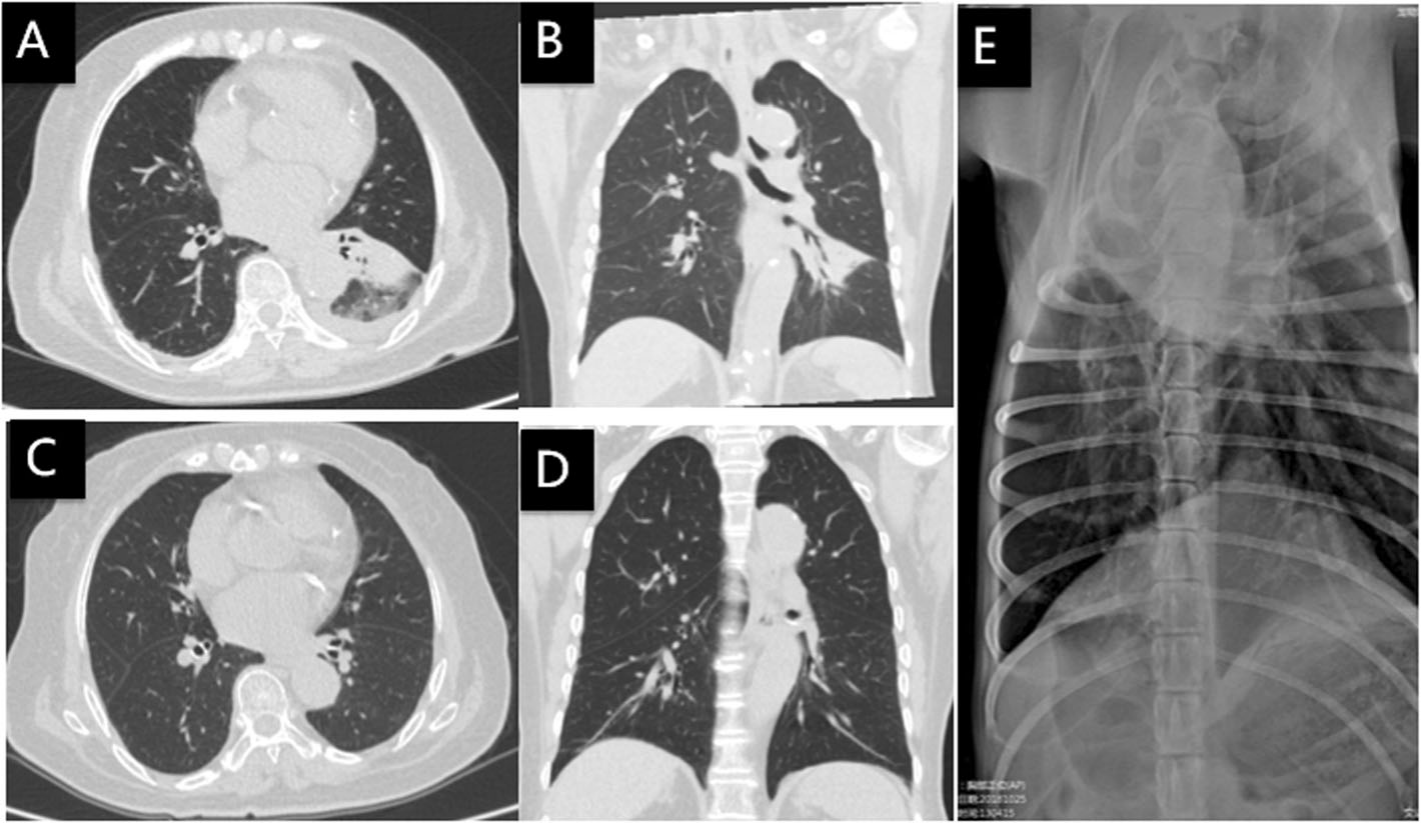
Chest CT and X-ray of case 1: **a**, **b** On the day of admission, **c**, **d** 21 days after doxycycline treatment, and **e** Chest X ray of patient’s pet dog

Body temperature upon arriving to us was 36.7 °C. Physical examination revealed inspiratory crackles in the lower left lung. CT and chest X-ray showed alveolar consolidation in the left lower lobe (Fig. 1a, b). White blood cell was largely normal: total count 7.71*10^9^/L (reference range: 3.5–9.5*10^9^/L), neutrophil count 5.38*10^9^/L (1.8–6.3*10^9^/L), 69.8% neutrophils (40–75%), 17.5% lymphocytes (20–50%), and lymphocytes 1.35*10^9^/L (1.1–3.2*10^9^/L). C-reactive protein (CRP) was 53 mg/L (0–8 mg/L). Erythrocyte sedimentation rate (ESR) was 74 mm/h (< 15 mm/h). Procalcitonin (PCT) was normal (< 0.05 ng/ml). Past history included hypertension, diabetes and coronary artery disease. Treatment with linezolid continued at 600 mg, I.V., q12h and meropenem was replaced with ertapenem (1 g, I.V., qd). Bronchoalveolar lavage (BAL) was conducted on the second day and BAL fluid (BALF) was sent for testing using mNGS (KindStar Global-Wuhan). On the 6th day of arrival to our hospital, mNGS reported sequence reads of *Chlamydia psittaci* (Table 1). Linezolid and ertapenem were discontinued. The patient was placed on doxycycline (100 mg, P.O., q12h). Symptoms gradually improved. CT scan and X-ray 21 days later showed radiological improvement (Fig. 1c, d). Upon close investigation, the patient disclosed pneumonia in her pet dog 2 days before her symptoms started (Fig. 1e). Treatment of the dog with ceftriaxone plus metronidazole was apparently not effective; pneumonia was cured with subsequent erythromycin.

### Case #2

**Fig. 2:**
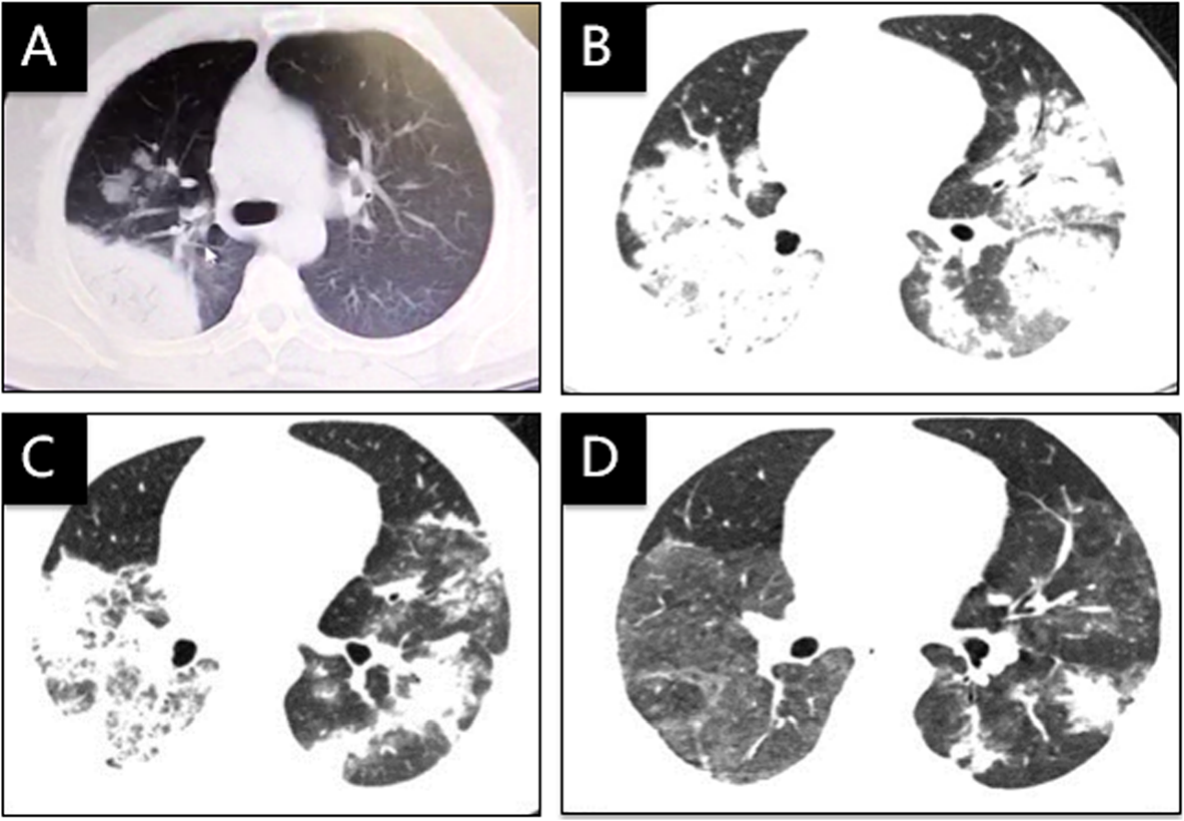
Chest CT of case 2: **a** On the 4th day of the onset, **b** On the 4th day of arrival to our hospital, **c** 7 days after treatment with doxycycline plus moxifloxacin, and **d** 3 weeks later after treatment with doxycycline plus moxifloxacin

A 45-year-old man was transferred to us with fever, productive coughing, generalized muscle ache and malaise for 7 days. On the 4th day of the onset, he visited a local hospital. A chest CT scan showed consolidation in the right upper lobe (Fig. 2a). Test results included: WBC count 8.22*10^9^/L, neutrophil count 7.65*10^9^/L, 93.1% neutrophils, 4.0% lymphocytes (count 0.33*10^9^/L), CRP 226 mg/L, ESR 42 mm/h, and PCT 1.712 ng/ml. A de novo diagnosis of diabetes was also established based on repeated testing of fasting blood glucose and hemoglobin A1c. Treatment with ceftazidime (2 g, I.V., q8h) and levofloxacin (500 mg, I.V., qd) was initiated for 3 days, but symptoms did not dissipate.

Upon transferring, the body temperatures was 39.5 °C, with crackles in both lungs. The patient received ertapenan (1 g, I.V., qd) and oseltamivir (75 mg, P.O., q12h), and fever subsided in 3 days. One days later, however, he developed high fever again (40 °C), with signs of respiratory failure (PaO_2_ 40 mmHg). On the 4th day of arrival to our hospital, a chest CT scan showed bilateral diffuse infiltration (Fig. 2b).

**Fig. 3:**
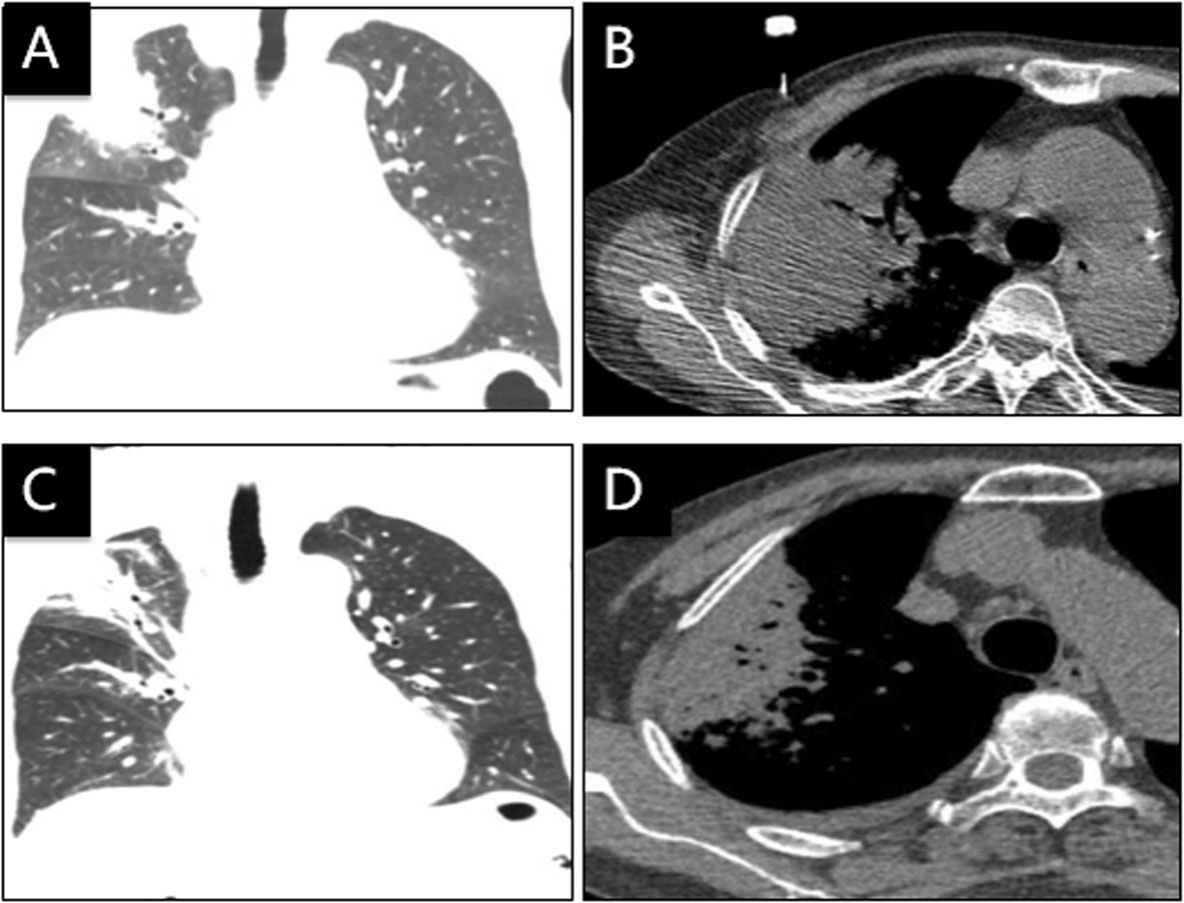
Chest CT of case 3: **a**, **b** On the day of admission, **c**, **d** 9 days after treatment with doxycycline plus moxifloxacin

BAL was conducted at this point. BALF analysis with mNGS (BGI-Shenzhen) revealed *Chlamydia psittaci* as well as a few other potential pathogens (Table 1). A close inquiry yielded a 20-year history of pigeon-farming. Ertapenan and oseltamivir were discontinued. Doxycycline (100 mg, P.O., q12h) plus moxifloxacin (0.4 g, I.V., qd) was initiated. The symptoms gradually subsided. CT scans 7 days later (Fig. 2c) and 3 weeks later (Fig. 2d) showed progressive infiltrate absorption.

### Case #3

An 85-year-old previously healthy woman presented with fever, productive coughing, headache, generalized muscle ache and emesis for 2 days. A chest CT scan showed consolidation in the right upper lobe (Fig. 3a, b). Laboratory test showed 6.38*10^9^/L WBC count, 88.9% neutrophils (absolute count: 5.67*10^9^/L), 8.0% lymphocytes (0.51*10^9^/L), 184 mg/L CRP, 64 mm/h ESR, and 1.64 ng/ml PCT.

Symptoms did not improve 3 days after ertapenem treatment (1 g, I.V., qd). Switching to biapenan (0.6 g, I.V., q12h) and teicoplanin (400 mg, I.V., qd) did not alleviate the symptoms. A percutaneous lung tissue biopsy was conducted; mNGS analysis (IngeniGen-Hangzhou) revealed infection with *Chlamydia psittaci* (Table 1). Treatment with doxycycline (100 mg, P.O., q12h) and moxifloxacin (0.4 g, I.V., qd) was initiated, and the patient recovered rapidly. A CT scan 9 days later showed partial absorption of the pulmonary infiltrate (Fig. 3c, d). The patient recalled planting a vegetable plot where birds often gathered.

### Case #4

**Fig. 4:**
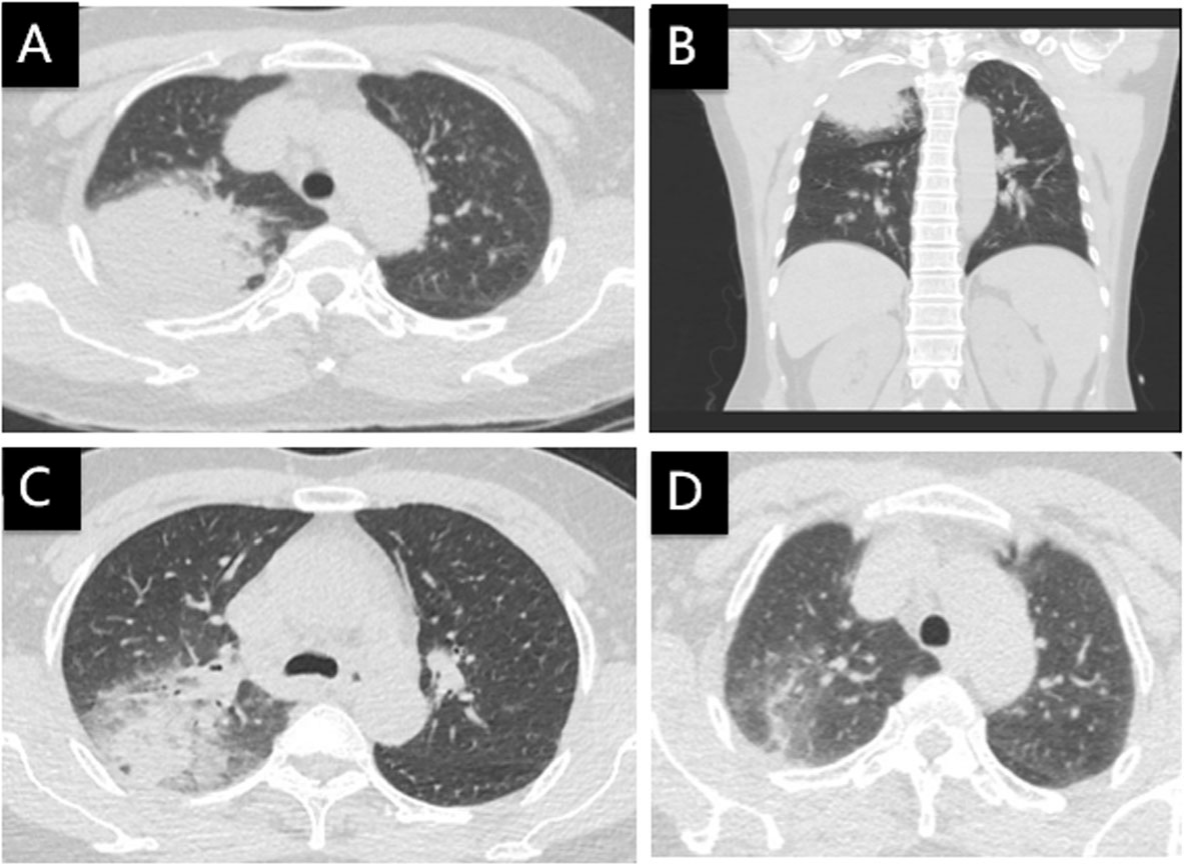
Chest CT of case 4: **a**, **b** On the day of admission, **c** Eight days after treatment with moxifloxacin, and **d**Sixteen days after discontinuation

A 66-year-old woman was transferred to us with fever, rigor, dry cough and dizziness for 6 days. At the beginning of the illness, she received levofloxacin and cefotaxime in a local hospital but symptoms persisted. A chest CT scan upon transferring showed consolidation in the right upper lobe (Fig. 4a, b). Laboratory test showed 5.47*10^9^/L WBC count, 69.8% neutrophils (3.82*10^9^/L), 19.4% lymphocytes (1.06*10^9^/L), 124 mg/L CPR, 17 mm/h ESR, and 0.13 ng/ml PCT. Treatment with moxifloxacin (0.4 g, I.V., qd) was initiated, and body temperature returned to normal within 2 days. A percutaneous lung biopsy was conducted; the sample testing with mNGS (IngeniGen-Hangzhou) detected *Chlamydia psittaci* (Table 1). Later, we learned that she had close contact with a large poultry farm on a daily basis. Moxifloxacin treatment lasted for 10 days. CT scan 8 days after moxifloxacin treatment initiation (Fig. 4c) and 16 days after discontinuation showed gradual infiltrate absorption (Fig. 4d).

### Case #5

**Fig. 5:**
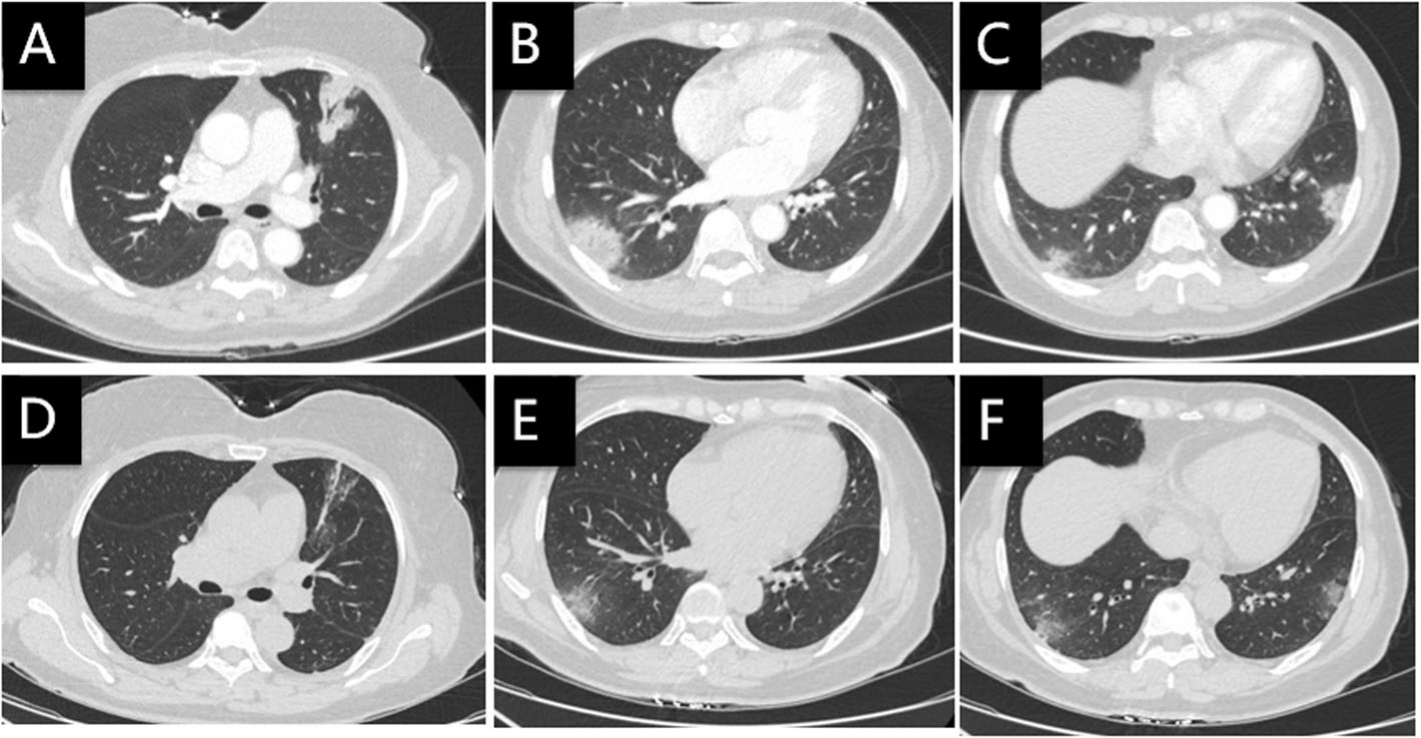
Chest CT of case 5: **a**, **b**, **c** On the day of admission, **d**, **e**, **f** Eight days after treatment with moxifloxacin

A 61-year-old woman presented with fever, rigor, weakness, productive coughing and dizziness for 3 days. A chest CT scan showed patchy infiltration and consolidation of both lungs (Fig. 5a, b, c). Laboratory test showed 6.88*10^9^/L WBC count, 68.4% neutrophils (4.70*10^9^/L), 22.7% lymphocytes (1.56*10^9^/L), 96.7 mg/L CRP, 37 mm/h ESR, and normal PCT. She disclosed close contact with a pet parrot. She was treated with moxifloxacin and her body temperature returned to normal on the next day. Analysis of lung biopsy tissue with mNGS (IngeniGen-Hangzhou) reported *Chlamydia psittaci* (Table 1). A CT scan 8 days later showed partial absorption of the pulmonary infiltrate (Fig. 5d, e, f).

## Discussion and conclusions

*Chlamydia psittaci* can be classified into 10 genotypes, with varying preference for host species [5]. Genotype A and E could infect humans. After entry via contaminated aerosols, *Chlamydia psittaci* spreads to the reticuloendothelial system. The lungs are the most common sites of *Chlamydia psittaci* infection. *Chlamydia psittaci* pneumonia is estimated to account for approximately 1% of community-acquired pneumonia cases [3, 6]. Symptoms mimics that of influenza, and typically include fever, fatigue, headache, myalgia, and coughing [7, 8].

In 4 out of the 5 cases in this report, patients had contacts with birds (parrot and pigeon) or poultry, suggesting the need to investigate such exposure upon suspected cases. Two out of the 5 patients had diabetes; and the other 3 were otherwise healthy, suggesting that *Chlamydia psittaci* could infect human subjects regardless of underlying diseases.

*Chlamydia psittaci* infection tends to be overlooked due to relatively low awareness by physicians. Laboratory testing for *Chlamydia psittaci* includes culture, serological assay, and PCR. Culture is time consuming, and most formidably, requires P3 facility [6]. Serological tests are only appropriate for retrospective diagnosis because sera from the both acute and convalescent phase of the illness are required [9]. PCR-based testing is the most specific and fastest method but only sensitive in the acute phases of the infection [2]. The current case series indicated that mNGS could be used to diagnose *Chlamydia psittaci* infection. If using a set of universally accepted standards, mNGS could even provide semiquantitative information (based on sequence reads) about the load of *Chlamydia psittaci*, such information could be critically important in determining whether a specific microbe is the causative pathogen(s) in polymicrobial samples. The limitations of mNGS analysis, for infection with a *Chlamydia psittaci* or any other agent, include host background sequences. In future studies, targeted sequencing and host depletion methods could be used to minimize the human host background, workflow quality control procedures could be optimized to reduce false positives. It is also likely that *Chlamydia psittaci* is merely present in the sample and not the culprit of infection. The fact that many other pathogens, including *Candida albicans*, have been identified in the 5 cases illustrate the complexity and a need to integrate the mNGS results into the overall clinical scenario. Lack of verification with serologic tests and/or culture is a significant limitation in the current study. These findings therefore must be interpreted with caution.

In the current series, mNGS was conducted only after initial empirical antibiotic treatment failed to control the infection in 4 out of the 5 cases. Considering the feasibility and cost of mNGS, we believed the timing of mNGS is appropriate, and recommend mNGS testing only if patients do not respond to treatments against other more common causes.

The specific reads for *Chlamydia psittaci* ranged from 2 to 225 in the 5 cases. Comparison across cases is not possible since the assay was conducted by several different companies. In case #1 and #5, the detected *Chlamydia psittaci* reads were 6 and 2, respectively. We speculate that the relatively low specific reads of Chlamydia psittaci in Case #1 may reflect the therapeutic effects of moxifloxacin, albeit not adequate. In Case #5, the low specific reads of *Chlamydia psittaci* may reflect the loss of biological activity of pathogens and degradation of nucleic acid during the process of sample collection and transportation or the treatments that the patient received prior to sample collection.

Recommended treatment for *Chlamydia psittaci* pneumonia included tetracycline, macrolide and quinolones [10]. Treatment must continue for at least 10–14 days to prevent relapse. In case #1, moxifloxacin was used as an initial treatment for 3 days, but no significant improvement was obtained. In our opinion, the lack of response to moxifloxacin in this case could be due to several reasons, including: 1) possible superinfection by other agents that could be readily controlled by doxycycline; 2) relative insensitivity of the *Chlamydia psittaci* isolate in this specific case to moxifloxacin. Indeed, recommended first-line treatment of *Chlamydia psittaci* pneumonia is doxycycline and not quinolones [11].

In conclusion, the current series suggested that mNGS could be used in diagnosing *Chlamydia psittaci* infection. This preliminary finding should be examined with diagnostic trial in the future.

## Data Availability

All the data supporting our findings is contained within the manuscript.

## Abbreviations

BAL: Bronchoalveolar lavage
BALF: Bronchoalveolar lavage fluid
CRP: C-reactive protein
CT: Computed tomography
ESR: Erythrocyte sedimentation rate
LN: Lymphocyte
mNGS: Metagenomic next-generation sequencing
NE: Neutrophil
PCR: Polymerase chain reaction
PCT: Procalcitonin
WBC: White blood cell

## References

[1] de Gier B, Hogerwerf L, Dijkstra F, van der Hoek W (2018). Disease burden of psittacosis in the Netherlands. Epidemiol Infect.

[2] Nieuwenhuizen AA, Dijkstra F, Notermans DW, van der Hoek W (2018). Laboratory methods for case finding in human psittacosis outbreaks: a systematic review. BMC Infect Dis.

[3] Hogerwerf L, DE Gier B, Baan B, Van Der Hoek W (2017). Chlamydia psittaci (psittacosis) as a cause of community-acquired pneumonia: a systematic review and meta-analysis. Epidemiol Infect.

[4] Gu W, Miller S, Chiu CY (2019). Clinical metagenomic next-generation sequencing for pathogen detection. Annu Rev Pathol.

[5] Radomski N, Einenkel R, Muller A, Knittler MR (2016). Chlamydia-host cell interaction not only from a bird's eye view: some lessons from chlamydia psittaci. FEBS Lett.

[6] Balsamo G, Maxted AM, Midla JW, Murphy JM, Wohrle R, Edling TM (2017). Compendium of measures to control chlamydia psittaci infection among humans (psittacosis) and pet birds (avian Chlamydiosis), 2017. J Avian Med Surg.

[7] Branley JM, Weston KM, England J, Dwyer DE, Sorrell TC (2014). Clinical features of endemic community-acquired psittacosis. New Microbes New Infect.

[8] Homma T, Yamaguchi T, Komatsu N, Hashimoto S, Doki Y, Senda K (2011). A case of acute psittacosis with severe abdominal pain. J Med Microbiol.

[9] Tuuminen T, Palomaki P, Paavonen J (2000). The use of serologic tests for the diagnosis of chlamydial infections. J Microbiol Methods.

[10] Cilloniz C, Torres A, Niederman M, van der Eerden M, Chalmers J, Welte T (2016). Community-acquired pneumonia related to intracellular pathogens. Intensive Care Med.

[11] Mandell LA, Wunderink RG, Anzueto A, Bartlett JG, Campbell GD, Dean NC (2007). Infectious Diseases Society of America/American Thoracic Society consensus guidelines on the management of community-acquired pneumonia in adults. Clin Infect Dis.

